# Chronic Hydroxyurea Therapy in Children with Sickle Cell Anemia: Mechanisms of Action, Systemic Effects, and Long-Term Safety

**DOI:** 10.3390/jcm14238599

**Published:** 2025-12-04

**Authors:** Federica Fogliazza, Martina Berzieri, Giulia Carbone, Davide Ciriaco, Susanna Esposito

**Affiliations:** Pediatric Clinic, Department of Medicine and Surgery, University of Parma, 43126 Parma, Italy; federica.fogliazza@studenti.unipr.it (F.F.); martina.berzieri@unipr.it (M.B.); giulia.carbone@unipr.it (G.C.); davide.ciriaco@unipr.it (D.C.)

**Keywords:** hydroxyurea, sickle cell anemia, sickle cell disease, fetal hemoglobin, neuroprotection, inflammation, long-term safety

## Abstract

Sickle cell disease (SCD) is the most common monogenic disorder worldwide and remains a major cause of morbidity and mortality. Sickle cell anemia (SCA), the homozygous HbSS genotype, represents the most severe and frequent form within the spectrum of SCD. Hydroxyurea (HU), a ribonucleotide reductase inhibitor, represents the first and most widely used disease-modifying therapy for SCA. This review summarizes current evidence on the mechanisms of action, clinical efficacy, systemic effects, and long-term safety of chronic HU therapy in patients with SCA. A comprehensive literature search was conducted in PubMed up to 2025 using the terms “sickle cell disease,” “sickle cell anemia”, “hydroxyurea,” and “children” or “paediatric.” Eligible studies included randomized controlled trials, cohort studies, and systematic reviews evaluating HU therapy in SCA. Literature analysis showed that HU exerts pleiotropic effects by inducing fetal hemoglobin (HbF) synthesis, improving red blood cell deformability, reducing leukocyte and platelet counts, and enhancing nitric oxide bioavailability. These mechanisms lead to decreased vaso-occlusive crises, acute chest syndrome, transfusion requirements, and overall mortality. Beyond hematologic improvement, HU confers neuroprotective benefits, modulates inflammatory and immune pathways, and supports normal growth and endocrine development in children. Adverse events, primarily mild bone marrow suppression, are dose-dependent and reversible with appropriate monitoring. No evidence supports an increased risk of malignancy with long-term use. In conclusion, chronic HU therapy is a safe, effective, and multifaceted treatment that substantially improves survival and quality of life in patients with SCA. Early initiation and individualized dosing maximize its therapeutic benefits and help prevent irreversible organ damage.

## 1. Background

With a 2021 global incidence of 382 affected newborns per 100,000 live births, sickle cell disease (SCD) represents the most common monogenic disorder worldwide, and its global health burden continues to grow [1,2,3,4,5]. In 2021, over half a million babies were estimated to be born with SCD, marking a >40% increase in the global patient population over the past two decades. The number of individuals living with SCD rose from approximately 5.5 million in 2000 to nearly 7.75 million in 2021. Among children under five years of age, SCD ranked 12th for total mortality and 40th for cause-specific mortality in 2021 [4].

SCD encompasses a group of autosomal recessive hemoglobinopathies characterized by the tendency of erythrocytes to adopt an abnormal sickle shape, a process that underlies the disease’s pathophysiological events and clinical manifestations [1,6,7,8]. The disorder results from the inheritance of pathogenic β-globin alleles, at least one of which is the βS variant (p.Glu6Val). Disease expression requires a sufficient concentration of sickle hemoglobin (HbS, α2βS2 tetramers) to induce polymerization upon deoxygenation, leading to red blood cell (RBC) sickling and both extravascular and intravascular hemolysis [1,6,7,8,9,10,11].

Sickling obstructs microvascular blood flow, causing ischemia–reperfusion injury, while hemolysis releases erythrocyte damage-associated molecular patterns (eDAMPs) and reactive oxygen species (ROS), promoting endothelial dysfunction. These mechanisms establish a cycle of vaso-occlusion, tissue damage, and sterile inflammation that perpetuates disease progression. Environmental or inflammatory triggers such as infection, hypoxia, dehydration, and acidosis often initiate this cascade.

Clinically, SCD may manifest through acute complications—including vaso-occlusive crisis (VOC), acute chest syndrome (ACS), infections, stroke, and splenic sequestration—as well as chronic degenerative damage affecting multiple organs [7]. Disease phenotype correlates strongly with genotype: sickle cell anemia (SCA; homozygous βSβS), HbS/β-thalassemia (βSβ-thal), and HbS/C disease (βSβC) are the main genotypic variants [2,3,12,13,14,15,16,17,18]. SCA and HbS/β^0^-thalassemia typically present with more severe clinical and hematological features, whereas HbS/C and HbS/β^+^-thalassemia generally exhibit milder forms [19,20]. The severity of SCD varies widely and depends on both genetic factors—including the underlying genotype (such as HbSS, HbS/β^0^-thalassemia, HbS/β^+^-thalassemia, and HbSC) and fetal hemoglobin (HbF) levels—as well as non-genetic modifiers such as infection status, nutritional status, environmental exposures, access to care, and socioeconomic conditions [2,12,13,14,15,16,17].

Hydroxyurea (HU) therapy has become a cornerstone of disease-modifying treatment, particularly for patients with SCA and HbS/β^0^-SCD. HU effectively reduces the incidence of VOC, ACS, and cerebrovascular complications by increasing HbF production and improving blood rheology [21].

The present review aims to summarize and critically assess the outcomes of long-term hydroxyurea therapy in patients with SCA, focusing on its efficacy, safety, and systemic effects, based on the most recent scientific literature.

## 2. Methods

This manuscript is a narrative review, designed to provide a qualitative synthesis of current evidence rather than a systematic or quantitative analysis. The literature search was intentionally broad and not intended to generate an exhaustive dataset for statistical aggregation. Therefore, study characteristics were summarized descriptively within the text, without formal tabulation or quantitative analysis (e.g., by year, country, or study design).

An extensive literature review was conducted to evaluate the efficacy, safety, and systemic effects of chronic HU therapy in patients with SCA. The electronic search was performed in the PubMed database using combinations of the following keywords: “sickle cell disease” or “SCD” or “sickle cell anemia” and “hydroxyurea” and “children” or “paediatric”. Only articles published in English were considered. The search included studies published up to 2025 and encompassed randomized controlled trials, observational studies, meta-analyses, and systematic reviews. To ensure the inclusion of all relevant evidence, a manual search of the reference lists of eligible articles was also carried out.

Studies were included if they investigated the use of HU in paediatric patients with SCA (HbSS) and reported clinical outcomes such as VOC, ACS, transfusion requirements, neurological complications, or effects on growth and development. Research providing data on treatment safety and tolerability, including hematologic, renal, hepatic, and reproductive parameters, was also considered eligible for inclusion. Studies focusing exclusively on non-homozygous genotypes (for example, HbS/β-thalassemia or HbSC), as well as case reports, conference abstracts, or articles lacking sufficient clinical data, were excluded. Similarly, studies addressing experimental therapies not directly related to hydroxyurea were not included in the analysis.

Two reviewers (M.B. and D.C.) independently screened titles and abstracts retrieved from the initial PubMed search to identify potentially eligible studies. Full-text articles were then examined independently by the same reviewers to determine final eligibility according to the predefined inclusion and exclusion criteria. Any disagreements regarding study selection were resolved through discussion and consensus; when necessary, a third senior reviewer (G.C.) was consulted to adjudicate discrepancies.

Data extraction was performed using a standardized, pre-designed extraction form. Each reviewer independently extracted key variables from the included studies, including study design, population characteristics, sample size, treatment regimen, duration of follow-up, and primary clinical or laboratory outcomes. Extracted data were then compared, and any inconsistencies were resolved through joint review and consensus. When required, the senior author verified the extracted information to ensure accuracy and completeness.

## 3. Clinical Indications of Hydroxyurea Therapy in Children with Sickle Cell Anemia

According to the AIEOP (Associazione Italiana di Ematologia e Oncologia Pediatrica) guidelines, HU therapy is strongly recommended for pediatric patients with SCA who present with one or more clinically significant manifestations of the disease [21]. The main indications include frequent or severe VOC, recurrent or single severe episodes of ACS, dactylitis, pulmonary hypertension, and moderate-to-severe symptomatic chronic anemia. These complications reflect the recurrent vaso-occlusive and hemolytic events that drive the progressive organ damage and morbidity associated with SCA. HU acts as a disease-modifying agent by mitigating the underlying pathophysiologic mechanisms—chiefly by increasing fetal hemoglobin (HbF) production, improving blood rheology, and reducing inflammation and vascular occlusion.

A further indication for HU is primary stroke prevention in pediatric patients who have previously received at least one year of chronic transfusion therapy due to abnormal transcranial Doppler (TCD) velocities. HU may be introduced when magnetic resonance imaging (MRI) findings are normal and TCD velocities have normalized under careful clinical and instrumental monitoring [21]. In these cases, HU offers an effective and safer long-term alternative to chronic transfusions, reducing transfusion-related risks such as alloimmunization, iron overload, and transfusion-transmitted infections. In addition, HU can be employed for secondary stroke prevention in patients for whom chronic transfusion therapy is not feasible or is contraindicated.

Beyond these well-established indications, HU has also shown promise in several non-traditional or emerging clinical contexts. Among these are recurrent priapism, a painful and potentially disabling complication of SCA, and chronic leg ulcers (particularly malleolar ulcers), conditions for which HU may contribute to symptom control and improved healing [21]. Moreover, HU is sometimes considered in patients with frequent hospitalizations or school absences due to disease-related complications, as part of a broader strategy to enhance quality of life and reduce the psychosocial impact of SCA.

Recent evidence from American and international studies has further expanded the therapeutic scope of HU, emphasizing the importance of early initiation, even before the onset of clinical complications [22]. This paradigm shift is grounded in the understanding that SCA is a chronic progressive disorder in which end-organ damage begins in infancy, well before overt symptoms appear. The early use of HU, typically between 6 and 12 months of age, has been shown to reduce subclinical tissue injury, prevent irreversible organ damage, and improve long-term outcomes [22]. This preventive approach reflects a major evolution in disease management, aligning with a proactive, rather than reactive, therapeutic strategy.

While HU is broadly safe and well tolerated, certain contraindications must be carefully considered. HU should not be administered to individuals with significant renal impairment (serum creatinine > 2 mg/dL), advanced hepatic disease, or severe bone marrow suppression (absolute neutrophil count < 2000/mm^3^ and/or platelet count < 100,000/mm^3^). It is also contraindicated in pregnant or breastfeeding women, in patients with known hypersensitivity to HU, and in men and women of reproductive age not using effective contraception [21]. Close laboratory monitoring of blood counts and organ function is essential throughout treatment to ensure safety and to adjust dosing according to hematologic tolerance.

In pediatric patients, HU therapy is initiated at an oral dose of approximately 10 mg/kg/day for four weeks, followed by gradual escalation to the maximum tolerated dose (MTD), generally up to 35 mg/kg/day, under close hematologic monitoring [21]. Initially, due to concerns regarding cytotoxicity, its use in children was restricted to those with severe disease phenotypes. However, multiple clinical trials and long-term follow-up studies have demonstrated that HU’s toxicity profile in pediatric populations is comparable to that in adults, with manageable adverse effects and no significant long-term carcinogenicity [23,24,25,26,27]. Consequently, early and sustained HU therapy is now recommended as a cornerstone of disease-modifying treatment in both children and adults with SCA.

In summary, the clinical indications for HU have evolved from targeting only severe phenotypes of SCA to encompassing early, preventive, and long-term management strategies aimed at modifying disease progression. Current evidence supports HU as a cornerstone therapy for nearly all patients with SCA, provided that appropriate monitoring and individualized dose titration are implemented to maximize therapeutic benefits and minimize toxicity.

## 4. Mechanism of Action of Hydroxyurea

**Figure 1:**
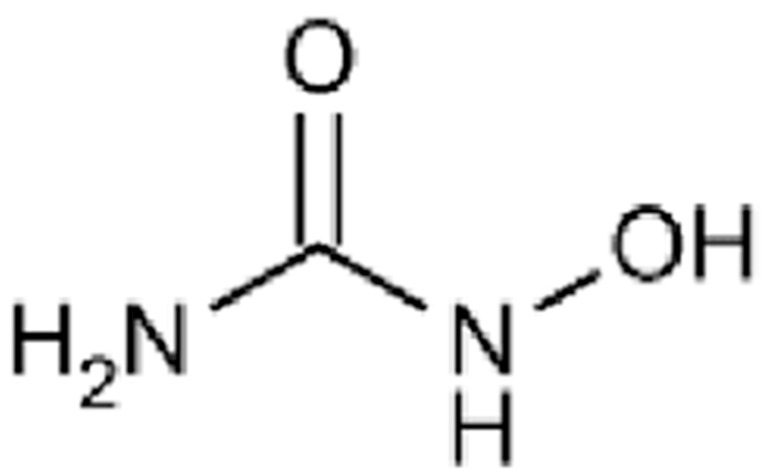
Chemical structure of hydroxyurea.

HU (Figure 1) is a hydroxylated urea derivative that acts primarily as an inhibitor of the enzyme ribonucleotide reductase, a key enzyme responsible for the conversion of ribonucleotides into deoxyribonucleotides, which are essential precursors for DNA synthesis [21].

By blocking this enzyme, HU exerts a cytostatic effect on rapidly dividing cells, leading to temporary inhibition of DNA replication and cell cycle arrest at the S phase. Owing to this mechanism, HU has long been used as a chemotherapeutic and cytoreductive agent in the management of hematologic malignancies, such as chronic myelogenous leukemia, and in myeloproliferative disorders like polycythemia vera and essential thrombocythemia [22].

In the context of SCA, HU’s therapeutic benefit extends beyond its cytotoxic properties, encompassing a range of pleiotropic hematologic, vascular, and molecular effects. The most prominent and well-characterized mechanism is the induction of HbF synthesis. HU stimulates HbF production by reactivating γ-globin gene expression in erythroid precursor cells. Its cytotoxic action induces mild bone marrow stress, which leads to compensatory erythropoietin release and expansion of erythroid progenitors with higher HbF production potential [28,29]. Increased HbF levels dilute intracellular concentrations of HbS and inhibit its polymerization under deoxygenated conditions, thereby preventing RBC sickling [21,30,31,32].

Beyond HbF induction, HU exerts significant effects on RBC rheology and membrane properties. It enhances erythrocyte deformability, reduces cellular density, and decreases hemoglobin concentration per cell, all of which improve microcirculatory flow and oxygen delivery [28,29]. Moreover, HU downregulates the expression of key adhesion molecules—including CD36, VCAM-1, and very late antigen-4 (VLA-4)—on reticulocytes and endothelial cells, thereby mitigating abnormal cell–cell interactions and adhesion to the vascular endothelium. This effect contributes to the reduction in VOCs that characterize SCD [28].

Another critical mechanism of HU involves its myelosuppressive and anti-inflammatory properties. By reducing leukocyte and platelet counts, HU decreases the number of circulating cells that contribute to vascular obstruction and inflammation [30]. Leukocytes, particularly neutrophils, play a crucial role in the pathogenesis of VOC by adhering to the endothelium and releasing proinflammatory cytokines and ROS. Thus, the moderate and controlled myelosuppression induced by HU improves blood viscosity, diminishes endothelial activation, and helps to restore vascular homeostasis [30].

HU has also been shown to enhance nitric oxide (NO) bioavailability, further contributing to vasodilation and endothelial protection [21,31]. HU undergoes metabolic conversion to NO or stimulates its endogenous release, leading to increased cyclic guanosine monophosphate (cGMP) production within vascular smooth muscle cells. This pathway not only improves vascular tone and blood flow but may also participate in HbF induction through cGMP-mediated signaling.

Taken together, these mechanisms highlight the pleiotropic nature of HU, acting synergistically at multiple biological levels to ameliorate the complex pathophysiology of SCD. The increase in HbF remains the most clinically relevant effect, as it directly reduces HbS polymerization and the consequent hemolysis, vaso-occlusion, and end-organ ischemia [21,32]. The overall clinical outcome of these combined effects includes a reduction in the frequency of painful crises, episodes of ACS, transfusion requirements, and long-term organ damage, ultimately decreasing morbidity and premature mortality.

**Figure 2:**
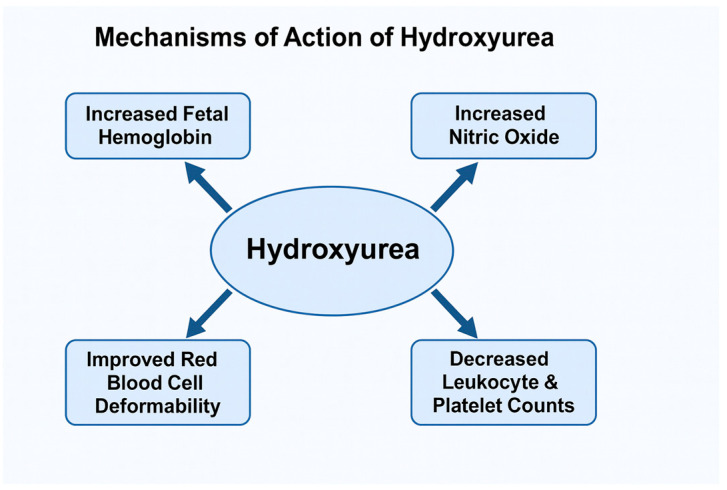
Effects of Hydroxyurea Action.

Figure 2 summarizes the effects of hydroxyurea action.

## 5. Non-Hematological Effects of Hydroxyurea Therapy in Children with Sickle Cell Anemia

The mechanism of action of HU extends well beyond its hematological effects, encompassing a broad spectrum of influences on multiple physiological systems. Its pleiotropic properties have been demonstrated to modulate not only erythropoiesis and vascular dynamics but also neurological, immunological, and endocrinological functions. These systemic effects contribute substantially to the overall clinical benefits observed in patients with SCA, underscoring HU’s role as a comprehensive disease-modifying therapy rather than a purely hematologic agent.

### 5.1. Neurological Effects

Neurological complications represent one of the most devastating consequences of SCA. Both acute and chronic cerebral injuries are common, including overt ischemic and hemorrhagic stroke, silent cerebral infarction (SCI), and neurocognitive impairment. It is estimated that up to one-third of individuals with SCA experience SCI by adulthood, while approximately 10% of children suffer an overt stroke before the age of 20 [28]. SCIs, though often clinically silent, are strongly associated with impaired executive function, reduced processing speed, and poorer academic performance [29]. These deficits typically emerge in early childhood, persist into adulthood, and are compounded by environmental and socioeconomic factors that exacerbate cognitive decline.

The neuroprotective effects of HU derive from several interrelated mechanisms. By inducing HbF production and increasing total hemoglobin concentration, HU improves blood oxygen-carrying capacity and cerebral perfusion, mitigating the chronic hypoxia that contributes to neuronal injury. Furthermore, HU reduces leukocytosis, thrombocytosis, and hemolysis, thereby lowering blood viscosity and microvascular obstruction in the brain. It also decreases oxidative stress and inflammatory signaling, leading to improved endothelial function and microvascular integrity [30]. Through these combined effects, HU may prevent or even partially reverse functional brain abnormalities and attenuate neurocognitive decline.

Multiple studies have evaluated HU’s impact on cognitive and neurological outcomes in pediatric populations with SCA [23,24,25,31]. Heitzer et al. demonstrated a significant association between HU therapy and higher performance scores across a range of neuropsychological assessments, including measures of working memory and attention [31]. Similarly, Wang et al. reported notable improvements in reading comprehension and overall academic performance among HU-treated children [23]. MRI and spectroscopy studies have also provided objective evidence of HU’s neuroprotective potential, showing reduced progression of white matter lesions and improved cerebral oxygen metabolism in treated patients [24,25].

Lai et al. used functional MRI to investigate HU’s effects on working memory networks, which are particularly vulnerable to hypoxia and vascular injury in adolescents with SCA. Their results revealed that HU therapy helps maintain activation patterns in prefrontal and parietal regions, supporting the preservation of cognitive function [26].

The SPRING trial further strengthened the evidence base for HU’s neurological benefits. This multicenter, randomized controlled study assessed HU’s role in primary stroke prevention, demonstrating that moderate-dose HU effectively reduces cerebrovascular risk without increasing toxicity or mortality. Moreover, moderate-dose HU was associated with significantly fewer hospitalizations for vaso-occlusive events and overall reduced healthcare utilization compared with low-dose regimens [27].

Similarly, the landmark TWiTCH trial, a phase III, multicenter, open-label, non-inferiority study, compared HU therapy with chronic transfusion in pediatric patients with abnormal TCD velocities but without severe vasculopathy. The results established HU as a non-inferior alternative to transfusion therapy for maintaining normal TCD velocities and preventing stroke recurrence [33]. This trial marked a paradigm shift in the management of cerebrovascular disease in SCA, positioning HU as a safe, effective, and cost-efficient long-term strategy for both primary and secondary stroke prevention.

### 5.2. Immunological Effects

SCA is increasingly recognized as a chronic inflammatory condition characterized by persistent immune activation and dysregulation. Historically, immune dysfunction in SCA was attributed primarily to functional asplenia, which impairs opsonization and clearance of encapsulated bacteria. However, accumulating evidence indicates that immune abnormalities in SCA involve more complex mechanisms, including hyperactivation of innate and adaptive immune pathways, altered cytokine production, and enhanced leukocyte adhesion and migration [34,35,36].

This state of chronic inflammation contributes to the pathophysiology of vaso-occlusion, tissue injury, and increased susceptibility to autoimmunization and alloimmunization—especially in patients undergoing recurrent blood transfusions with mismatched human leukocyte antigen (HLA) or RBC antigens [35,36].

HU has demonstrated a modulatory effect on immune system activation in SCA. By reducing hemolysis and improving endothelial function, HU indirectly lowers the release of eDAMPs that perpetuate immune activation. Moreover, HU decreases neutrophil counts and suppresses proinflammatory cytokine expression, contributing to a more regulated immune milieu.

Zahran et al. investigated regulatory T cell (Treg) populations—key mediators of immune tolerance—in children with SCD. Their findings showed that while both HU-treated and untreated SCA patients had elevated Treg levels compared to healthy controls, the Treg frequencies were significantly lower in HU-treated patients [37]. This suggests that HU may help normalize immune homeostasis and mitigate the excessive immune suppression and dysregulation seen in untreated individuals. These immunological changes were paralleled by clinical improvements, including fewer vaso-occlusive crises and acute chest syndrome episodes [38,39].

Furthermore, HU may attenuate oxidative stress-induced endothelial injury and decrease the expression of adhesion molecules on leukocytes and endothelial cells, thereby reducing vascular inflammation and thrombogenesis. Collectively, these effects position HU not only as a hematologic modulator but also as an immunoregulatory agent, capable of dampening the chronic inflammatory cycle that drives SCA pathology.

### 5.3. Endocrinological Effects

Chronic anemia and recurrent hypoxia in SCA can profoundly affect growth, endocrine function, and pubertal development. Prolonged hypoxemia and systemic inflammation interfere with the growth hormone (GH)–insulin-like growth factor 1 (IGF-1) axis, impairing bone growth and delaying puberty. Reduced hepatic synthesis of IGF-1, diminished GH secretion, and altered gonadotropin release contribute to these growth disturbances [40,41].

The impact of HU on growth and endocrine parameters has been a focus of pediatric research. The HUG-KIDS trial evaluated growth outcomes in children with SCA treated with HU at the maximum tolerated dose (MTD) for one year, comparing results with data from the Cooperative Study of Sickle Cell Disease (CSSCD) cohort. The study demonstrated that HU therapy did not adversely affect height, weight gain, or pubertal progression, alleviating earlier concerns regarding potential growth inhibition [42,43].

Mechanistically, the improvements in hemoglobin concentration and oxygen delivery observed during HU therapy likely enhance tissue perfusion and nutrient utilization, thereby supporting normal growth trajectories. Moreover, by reducing hemolysis and chronic inflammation, HU may preserve energy and protein stores previously consumed by compensatory erythropoiesis and oxidative stress responses.

Long-term studies further support these findings. Wang et al. and Nagalapuram et al. observed that HU-treated children maintained normal growth velocity and pubertal milestones, comparable to those of healthy peers and superior to untreated SCA patients [42,43]. These results highlight HU’s role not only in disease modification but also in improving developmental outcomes, emphasizing the importance of early and sustained therapy to prevent irreversible endocrine and growth deficits.

In summary, the non-hematological effects of HU span multiple organ systems, extending its therapeutic impact far beyond hematologic improvement. By enhancing cerebral oxygenation, modulating immune balance, and preserving normal growth and endocrine function, HU emerges as a multifaceted agent capable of mitigating both acute and chronic complications of sickle cell disease. These systemic benefits reinforce its position as a cornerstone therapy in the long-term management of SCD across all age groups.

Table 1 summarizes the clinical benefits of HU in SCA.

## 6. Adverse and Toxic Effects of Hydroxyurea Therapy in Children with Sickle Cell Anemia

The most common hematological adverse effect associated with HU therapy is bone marrow hypoplasia, which reflects the drug’s intended cytostatic and myelosuppressive properties. This effect is dose-dependent, typically reversible upon temporary discontinuation, and rarely progresses to severe or irreversible marrow failure [44]. Mild bone marrow suppression is an expected pharmacologic outcome of HU treatment, as it contributes therapeutically to reducing the circulating levels of leukocytes and platelets—cell types that play key roles in the inflammatory and adhesive processes underlying vaso-occlusion in SCA [22].

In clinical practice, modest reductions in white blood cell (WBC) and platelet counts are common and generally desirable, as they improve blood rheology and decrease endothelial activation and inflammation. This controlled myelosuppression mitigates the formation of intravascular aggregates, thus reducing vaso-occlusive events and improving tissue oxygenation. However, careful monitoring is essential to prevent excessive cytopenia that could predispose patients to complications.

Importantly, neutropenia induced by HU does not appear to increase susceptibility to infection [45]. Several studies, including long-term pediatric and adult cohorts, have demonstrated that infection rates in patients receiving HU are comparable to those observed in untreated individuals. This is particularly relevant in the pediatric population, where earlier concerns regarding HU-induced immunosuppression have been largely dispelled by large multicenter trials, such as the BABY HUG and HUSOFT studies [46]. Therefore, HU therapy should not be discontinued for mild or moderate infections or during short febrile episodes, as temporary interruptions can reduce therapeutic efficacy and HbF levels.

Conversely, in cases of aplastic crisis—whether due to viral infections such as parvovirus B19 or other causes—HU should be suspended as a precautionary measure, regardless of etiology, until hematologic recovery is confirmed [21,46]. Routine and periodic complete blood count (CBC) monitoring is fundamental for all patients undergoing HU therapy. Laboratory assessments typically include measurement of hemoglobin, hematocrit, reticulocyte count, leukocyte differential, and platelet count, along with renal and hepatic function tests to guide safe dosing adjustments.

As a safety threshold, treatment should be temporarily withheld if the absolute neutrophil count (ANC) falls below 1500/μL, platelet count drops below 80,000/μL, or the absolute reticulocyte count declines below 80,000/μL in the context of hemoglobin < 9 g/dL [21]. Once counts recover, HU may be restarted at a lower dose or resumed at the previous dose if marrow suppression is deemed transient and dose-related.

Other systemic or related effects can accompany hematologic changes. Cutaneous manifestations, including hyperpigmentation of the skin and nails, xerosis, erythematous rashes, or ulcerations, have occasionally been reported but are typically mild and reversible upon dose adjustment or temporary discontinuation [47]. Renal excretion is the primary route of HU elimination, and mild, transient increases in serum creatinine have been observed in some patients; hence, periodic renal monitoring is recommended, particularly in individuals with pre-existing renal impairment [48].

Reproductive effects are an additional consideration in long-term therapy. Reversible oligospermia or asthenozoospermia has been described in male patients, while the impact on female fertility appears limited and less well characterized [48,49]. Despite these findings, no consistent evidence has emerged linking HU use to permanent infertility or teratogenic effects when used under appropriate medical supervision.

Safety in pregnancy remains uncertain due to concerns about the increased risk of congenital malformations or abnormal growth [50].

Overall, the hematological adverse events of HU are predictable, dose-dependent, and largely manageable through regular clinical and laboratory surveillance. The risk of serious complications is minimal when therapy is properly monitored. Notably, despite initial concerns, no evidence has been found to support an increased incidence of leukemia or other malignancies in SCA patients treated with HU [21]. The balance of evidence strongly supports that the hematologic benefits of HU—particularly its capacity to raise HbF and reduce vaso-occlusive burden—far outweigh its potential risks when administered and monitored appropriately.

Table 2 describes the adverse effects of HU.

## 7. Conflicting Evidence and Sources of Heterogeneity

Although the majority of the available literature supports the efficacy and safety of chronic HU therapy in children with SCA, certain outcomes present heterogeneous or conflicting findings. Differences in study design, patient characteristics, follow-up duration, and outcome definitions contribute substantially to these divergences.

Most long-term pediatric and adult cohorts report no increased incidence of leukemia or other malignancies with HU treatment [21,44,48]. These supportive findings typically originate from prospective studies with sustained monitoring and robust follow-up.

In contrast, occasional reports suggesting a malignancy signal arise from small case series or from populations with substantial confounders such as prior transfusions or comorbid cytopenias. Such discrepancies likely reflect the background inflammatory and hemolytic burden of SCA rather than HU exposure itself [44,48].

Evidence regarding HU’s effects on fertility—particularly in males—remains mixed. Supportive studies indicate that HU-related oligospermia is dose-dependent and generally reversible after treatment interruption, with improvements in sperm concentration and motility documented in longitudinal follow-up [48]. However, the reversibility timeline varies, and interpretation is complicated by the fact that SCA itself impairs spermatogenesis through chronic hypoxia, oxidative stress, recurrent testicular vaso-occlusion, and iron overload [49]. These disease-related mechanisms often make it difficult to distinguish HU-induced changes from the underlying reproductive dysfunction associated with SCA.

Less supportive studies report persistent abnormalities in semen parameters, although these findings often arise from cross-sectional designs, small samples, or lack of pre-treatment baselines. Heterogeneity is driven by variations in patient age, disease severity, HU dose and duration, and inconsistent laboratory methods used for sperm evaluation.

In contrast, evidence on female fertility remains very limited. No robust prospective studies have assessed HU’s effects on ovarian reserve, menstrual regularity, or long-term reproductive outcomes. While available data do not show a clear association between HU and impaired ovarian function, the lack of systematic hormonal assessments (e.g., AMH levels), ultrasonographic data, or pregnancy-related endpoints represents a major evidence gap. Interactions among HU exposure, chronic inflammation, and endocrine alterations in SCA also remain insufficiently understood.

Given these uncertainties, expert consensus supports pre-treatment fertility counseling for all adolescents and adults of reproductive age. For males, this includes offering baseline semen analysis and discussing the option of sperm banking in selected cases, especially before initiating long-term high-dose HU therapy. For females, clinicians should document menstrual and reproductive history, consider ovarian reserve testing where feasible, and ensure longitudinal follow-up. Such counseling allows families and patients to make informed decisions while acknowledging the limitations of current evidence.

Several prospective studies and imaging-based trials demonstrate that HU improves or stabilizes neurocognitive performance, reduces white-matter injury, and enhances cerebral metabolic efficiency [23,24,25,26,27,31,32].

However, some reports show no significant improvement, typically due to shorter follow-up intervals, limited neuropsychological batteries, or inclusion of children with pre-existing neurological injury [28,29,30]. Social factors—including socioeconomic status, educational environment, and comorbid learning difficulties—may also influence outcomes and contribute to heterogeneity.

Supportive studies, particularly from large multicenter cohorts, show that HU does not increase infection rates and may even reduce infection-related morbidity, largely by decreasing hemolysis and improving baseline health status [45,46].

Studies reporting higher infection rates often involve settings with low vaccination coverage, inconsistent antibiotic prophylaxis, or high background prevalence of malaria or bacterial infections, indicating that geographic and health-system factors, rather than HU itself, largely account for differences in infection risk [45].

## 8. Conclusions

Chronic HU therapy is a cornerstone of care for children with SCA, offering a well-established, disease-modifying effect across hematologic, vascular, and systemic pathways. By increasing HbF levels and improving RBC rheology, HU effectively reduces hemolysis, vaso-occlusive events, ACS, and transfusion requirements, leading to substantial reductions in morbidity and early mortality. Beyond its hematologic benefits, HU provides neuroprotective, immunomodulatory, and endocrinological advantages, with long-term studies confirming its safety and showing no evidence of increased carcinogenicity.

Although access, adherence, and clinician hesitancy continue to limit its optimal use in some settings, HU remains a safe, effective, and widely accessible therapy capable of significantly improving quality of life and long-term outcomes for children with SCA. Continued efforts to enhance early diagnosis, expand treatment availability, and generate long-term real-world data will be essential to support equitable and optimized care for all patients.

## Figures and Tables

**Table 1:** Clinical Benefits of Hydroxyurea (HU) in Sickle Cell Anemia.

Outcome	Evidence Summary	Clinical Impact	Evidence Strength (Trial vs. Cohort) + Key Sources
Reduction in vaso-occlusive crises	HU increases HbF and improves RBC rheology, reducing sickling and microvascular obstruction	Fewer pain crises and hospitalizations	Strong evidence from randomized trials and prospective cohorts [22,32,42]
Reduction in acute chest syndrome	HU reduces hemolysis, inflammation, and leukocyte adhesion	Lower ACS incidence and respiratory complications	Randomized controlled trials and long-term observational cohorts [21,22,32]
Prevention of stroke and silent cerebral infarction	HU maintains normal TCD velocities and improves cerebral oxygenation	Effective alternative to chronic transfusion for primary stroke prevention	Phase III randomized controlled trials and real-world cohorts [25,32,33]
Improved anemia and hemolysis markers	HU increases total Hb and reduces reticulocytes, LDH, and bilirubin	Improved oxygen delivery and lower hemolysis burden	Prospective trials and laboratory follow-up cohorts [21,22,30,42]
Reduced transfusion requirements	HU reduces VOC/ACS frequency and improves baseline Hb	Lower transfusion exposure and reduced iron overload risk	Randomized controlled trials and real-world cohort studies [22,32,33]
Neurocognitive preservation	Improved cerebral perfusion and reduced silent infarct progression	Better neurodevelopmental and cognitive outcomes	Prospective neurocognitive and neuroimaging studies [26,27,28,29,30,31,33]
Reduced inflammatory burden	HU decreases leukocytosis, cytokine activation, and endothelial adhesion	Lower systemic inflammation and vascular injury	Observational immunologic and inflammatory marker cohorts [21,34,39]
Normal growth and puberty	Improved oxygenation and metabolic balance	Supports normal growth velocity and pubertal development	Prospective pediatric trials and longitudinal cohorts [40,41,42,43]

Abbreviations: ACS, acute chest syndrome; Hb, hemoglobin; HbF, fetal hemoglobin; HU, hydroxyurea; LDH, lactate dehydrogenase; RBC, red blood cells; TCD, transcranial Doppler; VOC, vaso-occlusive crises.

**Table 2:** Adverse Effects of Hydroxyurea (HU), Frequency, and Management.

Adverse Effect	Frequency	Management	Notes
Bone marrow suppression (neutropenia, thrombocytopenia, low reticulocytes)	Common, dose-dependent	Temporary drug interruption and dose adjustment	Fully reversible with monitoring
Gastrointestinal symptoms (nausea, anorexia)	Occasional	Symptomatic treatment; take with food	Mild and transient
Cutaneous changes (hyperpigmentation, xerosis, nail changes)	Occasional	Emollients or dose modification if needed	Usually reversible
Renal or hepatic laboratory alterations	Rare	Periodic renal and liver monitoring	Usually mild and transient
Oligospermia in males	Occasional	Monitor if fertility concerns	Effects reversible after discontinuation
Aplastic crisis (e.g., parvovirus B19)	Rare	Suspend HU until count recovery	Not directly caused by HU
Teratogenic risk (pregnancy)	Rare	Avoid in pregnancy; use contraception	Contraindicated during pregnancy

Abbreviation: HU, hydroxyurea.

## Data Availability

All the available data are included in the manuscript.
